# The Benefit of Epidural Transforaminal Injection of Ozone in Comparison With Transforaminal Steroids Injection in the Management of Chronic Low Back Pain in Lebanese Population: One-Year Retrospective Study

**DOI:** 10.7759/cureus.34106

**Published:** 2023-01-23

**Authors:** Ammar Chemeisani, Hasan Tarhini, Thuraya Haj Ali, Ali A Jibbawi, Khalil Yehya, Ali Msheik

**Affiliations:** 1 Neurological Surgery, Lebanese University Faculty of Medicine, Hadath, LBN; 2 Medicine, Lebanese University, Hadath, LBN; 3 Neuroscience Research Center, Lebanese University Faculty of Medicine, Hadath, LBN; 4 Pediatrics, Rafik Hariri University Hospital, Beirut, LBN; 5 General Medicine, Lebanese University Faculty of Medicine, Hadath, LBN; 6 Neurological Surgery, Zahraa Hospital UMC, Lebanese University Faculty of Medicine, Beirut, LBN

**Keywords:** chronic pain management, acute pain, minimally-invasive spine, chronic low back pain (clbp), steriods, ozone therapy

## Abstract

Background

Chronic low back pain (CLBP) is one of the most common complaints affecting the population worldwide including in Lebanon. Until 15 years ago, surgery was the treatment of choice. However, conservative measures are now preferred because of the large number of post-surgical complications, in addition to the many conditions where surgery cannot be performed.

Objective

The aim of our study is to determine the effectiveness of transformational epidural injection of ozone (TFEOI) in the management of CLBP among the Lebanese population in the Nabatieh area in comparison with patients who received transformational epidural steroid injection (TFESI).

Methods

A one-year (2016-2017) retrospective study where 100 patients with CLBP were selected from two hospitals (Alnajdah, and Ragheb Harb hospitals) and divided into two groups. Fifty patients were treated with Ozone injections while the other 50 were treated with steroid injections. For each patient, we recorded the type of pain, irradiation, paresthesia, and the type of injection given (steroid or Ozone). We used the patients' files and contacted them via phone calls. The results of this study were reached based on Vas Score and Mac Nab criteria which are subjective questionnaires.

Results

The study showed that the TFESI was effective for a short duration (86% of results were excellent and good after one month of injection, but they decreased to 16% after six months). On the other hand, TFEOI was effective over both short and long duration (82% excellent and good after one month, 64% excellent and good after six months).

Conclusion

Results from this study provide that ozone injection has high benefits in the management of CLBP in the Lebanese population.

## Introduction

General presentation

Lower back pain with or without sciatic nerve involvement affects roughly 80% of the population at least once in their lifetime [1]. According to the National Household Health Expenditure and Utilization Survey (NHHEUS, 1999), the primary morbidity in Lebanon in the year 2009 was lower back pain [2]. According to the latest iteration of the Global Burden of Disease study, which tracks the prevalence of deaths and diseases worldwide, lower back and neck pain went from ranking twelfth as a cause of disability-adjusted life years (DALYs) globally in 1990 to fourth in 2015. In most countries, it was the leading cause of disability. DALYs from low back and neck pain increased by more than 17% from 2005 [3].

Chronic low back pain is an increasing problem worldwide. Many studies performed in USA and China showed that back and spine impairments were more common in women. Differences in acute presentation, management, or outcome have important medical and economic implications. A high body mass index and a lack of physical activity are both risk factors for back pain in a world where obesity and overweight have increased by 28% since the year 1980 in adults [3].

Definition of chronic low back pain

Chronic low back pain (LBP) is a common disorder involving the muscles, nerves, and bones of the back for more than three months. Pain can vary from a sudden sharp feeling to a dull, constant ache. Presentation differs according to the cause, so properly identifying and describing symptoms leads to more accurate diagnoses. Patients complain of a combination or a single symptom of the following: dull pain, referred pain to the buttocks, legs, and feet, and pain worsening after prolonged sitting. Pain that improves within six weeks with or without conservative treatment is considered acute while chronic pain persists for more than three months (7-12 weeks) [4,5].

There are no specific characteristics that confirm or ignore the diagnosis of discitis in lower back pain [6]. More typical features include persistent low back, groin, and/or leg pain. Patients may have experienced prior episodes of acute lower back pain. CLBP may be localized medially, mediolateral or foraminal in the back. Ohnmeiss et al. reported more detailed referral patterns during provocative discography [7]. Discogenic pain originating from L3/L4 level typically radiates to the front (anterior) side of the right, left, or both thighs. That originating from L4/L5 radiates to the outside (lateral) of the thigh and sometimes to the back (posterior) of the thigh. While that originating from L5/S1 usually causes pain on the back of the thigh or both sides of the thigh [8].

In the physical exam, most of the time there are not any typical characteristics of discogenic pain. Biphasic straightening from flexion is considered by some to be an indication of a disc complaint. Pain that radiates to the toes unilaterally or bilaterally is considered characteristic of discogenic low back pain. Vanharanta has described the pain radiating from the disc due to provocation with a tuning fork pressed on the processus spinosus of the affected segment [9]. Although suggestive, these physical examination characteristics have not been validated, and the current criterion standard for confirming a clinical diagnosis of discogenic pain is positive disc damage [10,11]. Therefore, we use imaging techniques such as computed tomography (CT) and magnetic resonance imaging (MRI), especially for detailed anatomical abnormalities in the vertebral column [12,13].

Chronic lower back pain origins may include injury, diseases, or prolonged stress on a specific part of the body [6]. CLBP may be classified as radicular or non-radicular according to the lesion place. Mostly associated with spinal nerve root irritation due to compression and inflammation is radicular. Non-radicular is due to abnormalities in facet joints, sacroiliac joints, and intervertebral disks [14]. In chronic low back pain, it is important to consider conservative treatment (non-surgical). If there was no improvement, the cause must be identified before choosing the most appropriate therapy.

Noninvasive imaging along with a good clinical examination (good history taking and physical exam) should be enough to identify or rule out underlying disease processes (fracture, malignancy, infection, and deformities), neurological disorders requiring surgical intervention (cauda equine syndrome, myelopathy), and psychological distress that may amplify or prolong the pain [15]. In patients with no neurological demonstrated deficit or a visible disk herniation at imaging, a spinal cause was identifiable in 15% [16].

Management of chronic low back pain

Different ways of treatment may be used in the management of chronic lower back pain. They include conservative nonpharmacologic treatment (physiotherapy), and pharmacologic treatment (non-steroidal anti-inflammatory drugs [NSAIDs], …), and for those with severe functional disabilities, radicular symptoms, or refractory pain, epidural therapeutic injections or even surgery may be preferred [17].

Conservative Treatment

Patients commonly use conservative treatment options with or without consulting the physician. Nonpharmacologic conservative treatment includes exercise therapy (focusing on strengthening core muscles), behavior therapy (relaxation), and acupuncture massage [17]. Acetaminophen is the first line of therapy because of its high safety profile. NSAIDs may also provide similar analgesia. In patients who did not benefit from either Acetaminophen or NSAIDS, opioids such as tramadol or other adjunctive medications may help [17].

Surgery

Surgery can be considered in patients with anatomic abnormalities consistent with the distribution of pain and patients with significant functional disabilities not improving even by nonsurgical treatments. Spinal fusion is used in treating back pain caused by fractures [14].

Epidural Therapeutic Injections

They include Ozone and steroids injection. Both techniques were found to have benefits for lower back pain [18]. Ozone (O3) is an allotropic form of oxygen, primarily known for its ecological properties, industrial application, and therapeutic effects [18]. Ozone is given via an injection into the herniated disc. When delivered, Ozone reduces the volume within the disc. This is due to the oxidation of proteoglycans, which are proteins found within the gel-like center of the disc (called the nucleus pulposus). When the disc volume is reduced, the pressure on nerves is also reduced. This in turn lowers the amount of pain. After many studies of the mixture of oxygen and Ozone gases, the combination was employed in medicine since the thirties in the treatment of different diseases, particularly in patients with thrombotic and ischemic diseases [19].

The benefit of using this gas mixture in the paravertebral muscles to treat pain has been studied. After many studies, researchers noted that the short-calculated oxidative stress achieved by ozone administration may correct a permanent imbalance caused by excessive or chronic oxidative injury. They discovered that repeated ozone treatment increases the activity of superoxide dismutase, catalase, and glutathione peroxidase, inducing a state of oxidative stress adaptation with major therapeutic implications. The mixture is produced by an apparatus (Ozone generator) that activates the molecules of diatomic oxygen in a voltaic arch [20]. Ultraviolet spectrophotometry allows precise quantification of Ozone percentages in the mixture obtained. In 1982, Jacobs reported the absence of side effects in over five million ozone therapy sessions for different pathologies [21]. The paravertebral intramuscular treatment produces pain relief in the majority of patients, together with decongestion, reabsorption of edema, and increased mobility. This has led to the idea of injecting the oxygen-ozone mixture into the intervertebral disc and conjugation foramen to obtain a powerful effect directly on the pathological mechanism [22].

Steroids are known to have an anti-inflammatory function. Steroid infiltration relieves the peri-ganglionic inflammation by ensuring recovery of the normal ganglio-neural myelin sheath, and hence nerve function, at the disease site [23]. During a trans-foraminal injection, a thin needle is inserted into the epidural space through the bony opening of the vertebral column from where a nerve root exits. The drug is delivered exactly in the area where the disc is compressing the nerve and causing inflammation when we injected steroids [24]. The procedure is performed with the patient lying on their belly using fluoroscopic guidance, which helps to prevent damage to the nerve root. A radio-opaque dye is injected to enhance the fluoroscopic images and to confirm that the needle is properly placed. This technique allows the steroids to be placed closer to the irritated nerve root. The exposure to radiation is minimal [24-26].

The procedure of injection of Ozone

Every patient will receive local anesthesia before the injection to avoid any pain. Then the patient lies with a pillow under his abdomen to correct lumber lordosis. This maneuver makes the lumber disc area approach to the base of the superior facet of the inferior vertebra, then the neurosurgeon uses a 15 cm long, 22 G spinal needle by directing it into the disc space at an angle of 50-60⁰ approximately. The angle can be obtained by oblique views. When the annulus is reached, the surgeon should twiddle a gritty feel of the annulus. Then the needle is pushed up to the center of the disc (nucleus pulposus) then with verifying the position with anterior, posterior, and lateral imaging. The tip of the needle should be in line with the spinous process and medial to the most medial aspect of the pedicle. The discography is done by using water-soluble contrast to confirm the needle position. When the disc is pathological, the dye spreads all along up to the canal and when the disc is normal, the dye remains in the center. Once confirmed, 5-7cc of 27-40mcg of epidural oxygen-ozone gas is injected into the disc space. The distraction of the space and whitish visualization of gas on the screen confirms the presence of gas in the disc space. The needle is removed, then a sterile dressing of the injection site should be done. The procedure lasts for approximately 20 to 30 mins and the patient is discharged home within two hours [26,27].

Studies on chronic low back pain

Women complain of lower back pain more than men do. This is probably due to their higher responsibilities as workers in addition to spending longer duration in household work and children care [27]. A recently proposed treatment for lumbar disk herniation is percutaneous intra-discal ozone injection. The effectiveness of this treatment has been tested in large clinical studies, findings of which have shown a positive outcome in 70%-80% of patients. In 2005, a study was accomplished by Gallucci et al., with the aim to prospectively compare the clinical effectiveness of intraforaminal and intradiscal injections of a mixture of a steroid and Ozone versus intraforaminal and intradiscal injections of a steroid and an anesthetic in the management of radicular pain related to acute lumbar disk herniation [28].

One hundred fifty-nine patients (86 men, 73 women; age range, 18-71 years) were included and were randomly assigned to two groups. Seventy-seven patients (group A) underwent intradiscal and intraforaminal injections of a steroid and an anesthetic, and 82 patients (group B) underwent the same treatment with the addition of Ozone. An Oswestry Low Back Pain Disability Questionnaire was administered before treatment and at several intervals, the last being at a six-month follow-up. Results were compared with the X2 test. The results showed that the intraforaminal and intradiscal injections of a steroid plus Ozone are more effective at six months than injections of only a steroid in the same sites [28].

Comparative studies between Ozone and steroid

Andreula Cosma and collaborators 2003 did a randomized control trial in Italy on 600 patients with lumbar disc herniation treated with a single session of Ozone therapy. All patients presented with clinical signs of lumbar disk nerve root compression with CT and/or MRI evidence of contained disk herniation. They randomized patients into two groups of 300 each. Group A received 4 mL intradiscal and 8 mL periganglionic Ozone at 27 µg/mL concentration. Group B received Ozone in a similar concentration plus steroid (1 mL Depo-Medrol 40 mg/mL) and 0.5% marcine. The outcome was assessed using modified Mac Nab criteria. Treatment was successful in 211 (70.3%) patients with excellent in 160 and good in 75 patients (p < 0.05). They concluded that Ozone therapy is a useful treatment for lumbar disk herniation that has failed to respond to conservative management and has a significant cumulative effect [29].

Shah et al. elaborated on a prospective study that was set up from 2006 to 2008. Patients with contained discs on MRI and failed conservative trials were included in the study. Those with cauda equina syndrome or spinal tumors or infections in addition to pregnant women were excluded. A total of 93 patients were injected with intra-discal ozone. There were 34 females and 59 males with an average age of 54±13.2 years (range 32 to 78). Fifty-one patients had two-level lesions while 24 had three-level lesions. Only 18 had single-level discs. Eleven patients had both sensorimotor deficits while six had only sensory and two had only motor deficits. The remaining patients had no neurological deficit. Eleven of these patients had previous open laminectomies done (failed back) [29].

Greenough and Fraser's score was used to study the outcome. The results showed that the mean follow-up was 27.86±3.25 months. The preoperative Greenough and Fraser score improved from a mean of 24.33±5.94 (12 to 45) to a postoperative (final follow-up) mean of 68.36±6.24 (50 to 75) (p < 0.001). Twenty patients had good results while 66 had excellent results according to Greenough and Fraser's score. Four patients did not follow up. Three patients had to undergo surgery in form of microdiscectomy. nine out of 13 patients with motor deficits had significant recovery (≥grade 4 power) while only three out of 17 having sensory symptoms continued to have sensory symptoms. Five patients in this series had post-operative headaches and were treated conservatively. There were no cases of infection. This study reports the successful treatment of contained lumbar disc herniation using intradiscal Ozone. However, a comparative and controlled trial will be essential to firmly establish this modality [30].

A study was done from June 2000 to December 2006 on the use of Ozone injection using a minimally invasive percutaneous technique for the treatment of lumbar disk herniation. The study was performed on 2,900 patients affected by lumbar disk herniation. Patients were selected on the basis of clinical, psychological, neurological, and neuro-radiological criteria. Exclusion criteria were extruded hernia and/or free disc fragments, hyperalgesia paralyzing sciatica, and progressive neurological impairment of the affected limb [30,31].

All patients were evaluated after one month, and those showing only partial success were scheduled for a second treatment session. The study’s results were evaluated with the modified Mac Nab classification, the visual analog scale (VAS), and the Oswestry Disability index at six and 12 months. Success rates were 75% to 80% for soft disc herniation, 70% for multiple disc herniation, and 55% for failed back surgery syndrome. None of the patients suffered early or late neurological or infectious complications. Based on these results, the researchers concluded that minimally invasive percutaneous treatment by Ozone infiltration is a valuable and competitive technique that provides excellent results at low cost and without complications [19].

## Materials and methods

Objectives

Previous studies and data analysis have demonstrated a considerable risk of failure and complications in both conservative and surgical treatment of chronic lower back pain, which raises concerns about seeking other treatment modalities with higher efficacy, less complication, and less risk of failure. In this study, we aim to evaluate the effectiveness of TFEO injection as an alternative treatment modality for patients with chronic lower back pain in the Lebanese population.

Primary Objective

The primary objective of our study is to determine the efficacy of TFEOI injection in the management of back pain in the NABATIEH area.

Secondary Objectives

We aimed to compare the results with the patients who received steroids injection, the results among the locations of radiculopathy, and the results among male and female patients.

Study design

This is a retrospective study targeting patients with CLBP who received a TFEOI or a TFESI in Al-Najdah and Sheikh Ragheb Harb hospitals in Lebanon in the area of Nabatieh. The duration of the study was from January 2016 to January 2017 (one year). In this study, we will compare the improvement in results between Ozone or steroid injections at three-time intervals (one month, three months, and six months).

Subjects and study population

One hundred patient files were randomly chosen from the files present in the two specified hospitals. All patients were diagnosed with CLBP (7-12 weeks) by clinical symptoms and MRI or CT-scan images. The patients were categorized according to the type of sciatica and they were divided into two groups: 50% (50 patients) were treated by transforaminal epidural ozone injection (intradiscal), and 50% (50 patients) received transforaminal epidural steroids injection. In Table 1, the inclusion and exclusion criteria are enlisted.

**Table 1:** The inclusion and exclusion criteria

Inclusion criteria	Exclusion criteria
Patients aged between 26 and 72 years old	Patients under 26 or over 72 years old
Patients who are positively diagnosed with CLBP with confirmed images (Ct or MRI) of the spine	Patients with cauda equine syndrome
Patients who received TFEOI or TFESI injections, with no benefits with conservative treatment	Patients with ankylosing spondylitis
	Pregnant women
	Patients with spinal tumors
	Patients with congenital musculoskeletal abnormalities

Data collection

For each patient, information was collected from their file about their name, age, gender, paresthesia’s disk, radiculopathy, presence of numbness, results of CT or MRI, and whether they received an Ozone or steroids injection and their clinical improvement after injection at one month, three months, and six months. The VAS score for pain and Mac Nab criteria were used in the assessment of pain based on questions. Each patient received a phone call asking them about their symptoms before and after the injection of ozone and steroids.

VAS Score

VAS (visual analog scale) for pain is a measurement instrument for subjective characteristics or attitudes that cannot be directly measured. It’s a continuous scale comprised of a horizontal (HVAS) or vertical (VVAS) line, usually, 10 cm (100mm) in length, anchored by two verbal descriptors, one for each symptom extreme, “no pain” (score of 0) and “pain as bad as it could be” or “worst imaginable pain” score of 10 [31]. It is demonstrated in the figure presented in the appendix.

Mac Nab modified Criteria

Mac Nab scale is used to determine the outcome assessment of patient satisfaction after ozone or steroids injection therapy [29]. The state of satisfaction was graded as excellent, good, fair, or bad. The excellent result means that the patient had no complaints and was able to return to full working capacity. The good result indicates that the patient had full working capacity but slight low back and leg pain. The fair result indicates that the patient does not have normal working capacity; low back and leg pain was reduced but the patient still required the administration of analgesics. Bad and no improvement results mean that the degree of pain is unchanged or worse.

Data statistical analysis

For data entry and analysis, we used the statistical package for social sciences for windows version 22 (IBM Corp., Armonk, NY). Independent samples t-test was used to test statistical significance. Descriptive analysis of quantitative variables will be presented as mean, standard deviation, minimum and maximum values. In addition, the software will also comprise the frequency and percentage of each category of patients. The number of missing values will be reported for each variable if present. A two-sided P-value of 0.05 was considered to be statistically significant.

## Results

Numbers of females and males who had both types of injections

**Figure 1:**
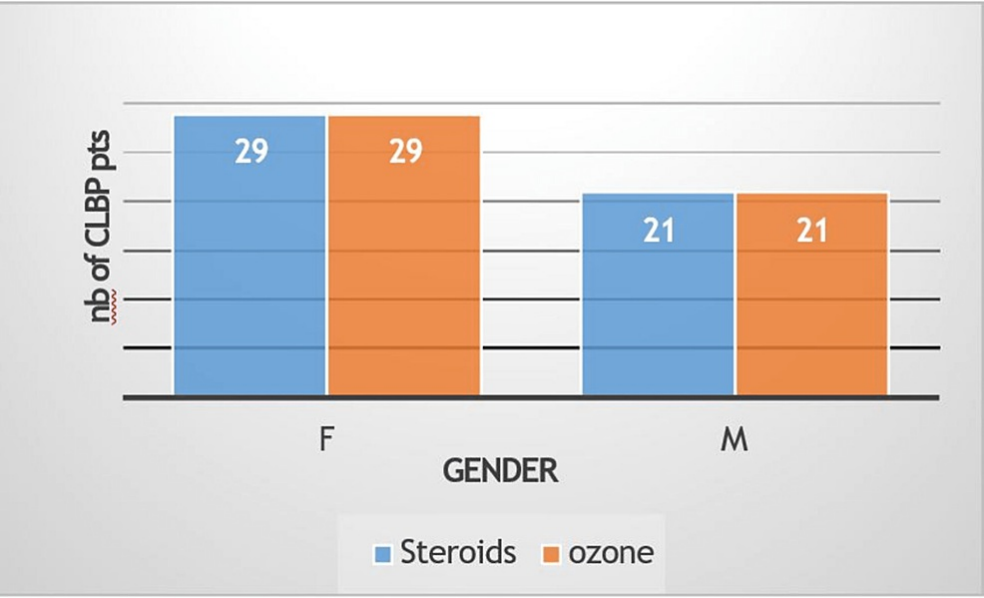
Histogram of the variations of gender according to both injections types F: female; M: male.

Fifty-eight patients of the total patients were females, and 42 patients were males. The mean age equals 49.86±12.664 years. The total number of patients is 100 patients, and the age range is 26-72 years (Figure 1).

Distribution of patients per type of sciatica, site of paresthesia in disc, and per location of radiculopathy

Table 2 represents the distribution of the study patients per type of sciatica, site of paresthesia in the disc, and location of radiculopathy. Concerning the type of sciatica, 40 patients had right sciatica, 37 patients had left sciatica, and 23 patients had bilateral sciatica. Concerning the site of paresthesia’s disc, 65 patients had medullolateral paresthetic disc, 20 patients had Medullar paresthetic disc, and 15 patients had foraminal paresthetic. Concerning the location of radiculopathy: 40 patients had S1 radiculopathy, 34 patients had L5 radiculopathy, 16 patients had L4 radiculopathy and 10 patients had L3 radiculopathy. The total number of patients is 100 (Table 2).

**Table 2:** Distribution of patients per type of sciatica, site of paresthesia in the disc, and per location of radiculopathy

Number of patients			Total number of patients
Per type of sciatica	Right	40	100
	Left	37	
	Bilateral	23	
Per site of paresthesia in the disc	Medullolateral	65	100
	Medullar	20	
	Foraminal	15	
Per location of radiculopathy	L3	10	
	L4	16	100
	L5	34	
	S1	40	

Results of VAS score before and after one, three, and six months of injection of steroids and ozone in females

**Figure 2:**
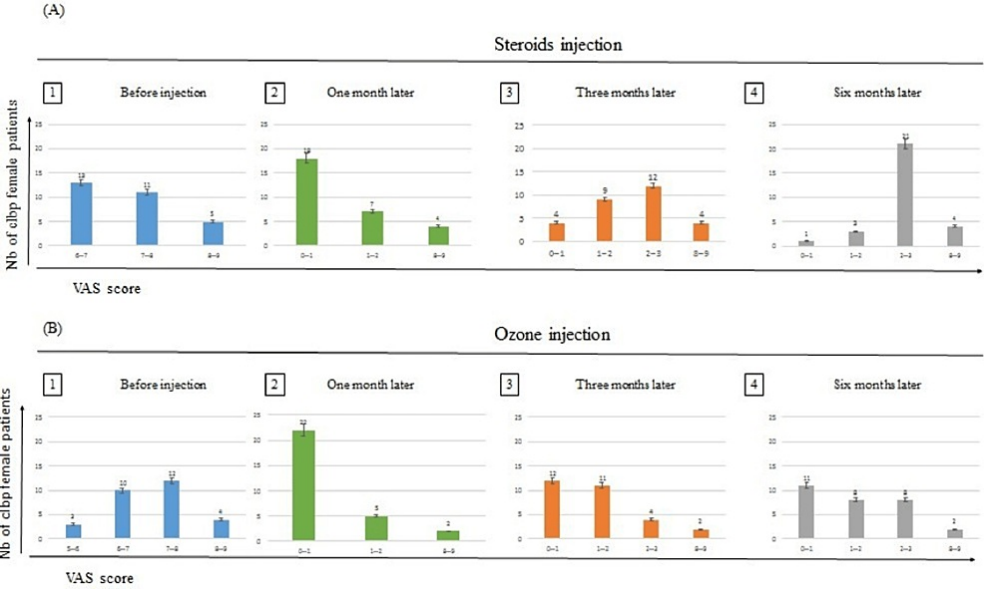
Comparison of steroids and ozone injections before and after one, three, and six months of treatment according to VAS score scale in female patients. The graph represents the number of patients and their response to either type of injection. This response is classified according to VAS score, with differences in the results between ozone and steroids injection after one month (p-value: 0.072), three months (p-value: 0.672), and six months (p-value: 0.001).

We evaluated the number of female patients with different VAS score before and after one, three, and six months of injection of steroids and ozone (Figure 2).

Before Injection of Steroids and Ozone

Figure 2 (A1; B1) shows that five female patients who had steroids injection versus four female patients who had ozone injection were with a VAS score of 8-9 before the injections. 11 female patients who had steroids injection versus 12 female patients who had ozone injection were with VAS score 7-8 before the injections. Thirteen female patients who had steroids injection versus 10 female patients who had ozone injection were with VAS score 6-7 before the injections. Three female patients who had ozone injections were with VAS score of 5-6 before injection. The total number of female patients who had steroids injection equals 29 patients, which is the same number of patients who had ozone injections.

Results After One Month of Steroids and Ozone Injection

Figure 2 (A2; B2) shows that four female patients had a VAS score of 8-9 with steroids injection versus two females with ozone injection. Seven female patients had a VAS score of 1-2 with steroids injection vs five females with ozone injection. Eighteen female patients had a VAS score of 0-1 with steroids injection vs 22 females with ozone injection.

Results After Three Months of Steroids vs Ozone Injection

Figures 2 (A3; B3) show that four female patients who had steroids injection versus two female patients who had ozone injection had a VAS score of 8-9. Nine female patients who had steroids injection versus 11 female patients who had ozone injection had VAS score 1-2. Four female patients who had steroids injection versus 12 female patients who had ozone injection had VAS score 0-1. Twelve female patients who had steroids injection versus four female patients who had ozone injection had a VAS score of 2-3.

Results After Six Months of Steroids Versus Ozone Injection

Figures 2 (A4; B4) show that four female patients who had steroids injection versus two female patients who had ozone injection had a VAS score of 8-9. Three female patients who had steroids injection versus eight female patients who had ozone injections had a VAS score of 1-2. One female patient who had steroids injection versus 11 female patients who had ozone injection had a VAS score of 0-1. Twenty-one female patients who had steroids injection versus eight female patients who had ozone injections had a VAS score of 2-3.

Differences between the results according to sample T-test in females after one, three, and six months of injections

We conducted an independent samples t-test (two-tailed, a= 0.05) to assess whether the effect of steroid injection differed significantly from Ozone injection after one month, three months, and six months. The assumptions of normality and equal variances were evaluated with no violations noted. There was no significant difference after one month where the p-value = 0.072 > 0.05 and after three months the p-value = 0.672 > 0.05 from the injection between Ozone and Steroids. However, there was a significant difference after six months of injecting (p =0.001 < 0.05) between results according to VAS score after six months of injection between female patients who had ozone injections and who had steroid injections (Table 3).

**Table 3:** Variations between ozone and steroids injections in female patients according to VAS score after one, three, and six months of injection N= the total number of female patients equals 29 for each type of injection

	Type of injection	N*	Mean	Std. Deviation	P-value
VAS score after one month	steroids injection	29	1.52	.738	0.072
	ozone injection	29	1.31	.604	
VAS score after 3 months	steroids injection	29 2.55		.910	0.672
	ozone injection	29	1.86	.915	
VAS score after 6 months	steroids injection	29	2.97	.626	0.001
	ozone injection	29	2.03	.981	

Results of VAS score before and after one, three, and six months of injection of steroids and ozone in male patients

**Figure 3:**
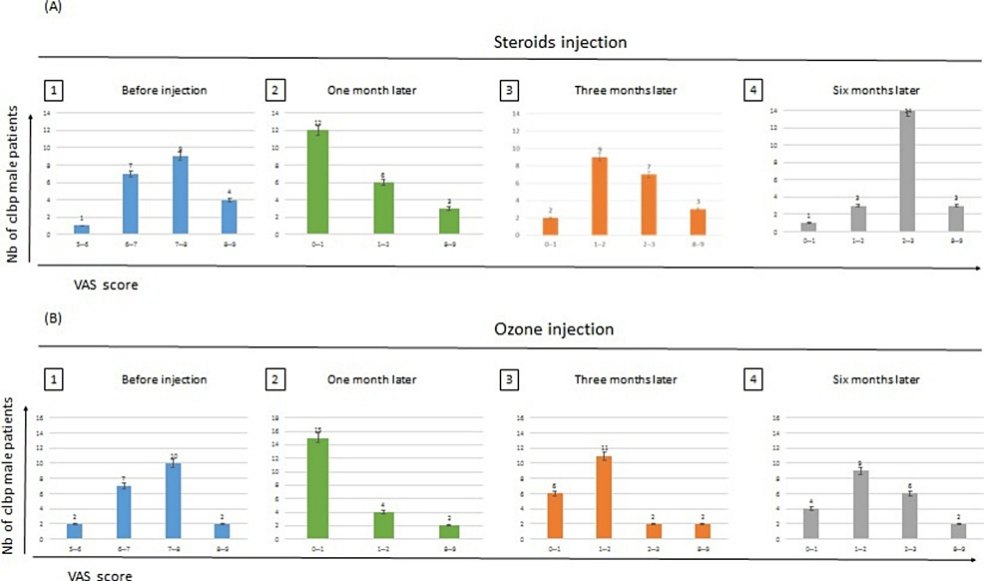
The graph represents the number of patients and their response to either type of injection. This response is classified according to VAS score, with differences in the results between ozone and steroid injections after one month (p-value: 0.321), three months (p-value: 0.344), and six months (p-value: 0.042). Comparison of steroids and ozone injections before and after one, three, and six months of treatment according to the VAS score scale in male patients

We evaluated the number of male patients with different VAS score before and after one, three, and six months of injection of steroids and ozone (Figure 3).

VAS Score Results Before Injection of Steroids and Ozone

Figures 3 (A1; B1) show that four male patients who had steroid injections versus two male patients who had ozone injection were with a VAS score of 8-9 before the injections. Nine male patients who had steroids injection versus 10 male patients who had ozone injection were with VAS score of 7-8 before the injections. Seven male patients who had steroids injection versus seven male patients who had ozone injection were with VAS score of 6-7 before the injections. One male patient who had steroids injection versus two male patients who had ozone injection were with VAS score 5-6 before injection. The total number of male patients who had steroids injection equal 21 male patients equal to 21 male patients who had ozone injections.

Results After One Month of Steroids Versus Ozone Injection

Figures 3 (A2; B2) show the results after one month between the patients who had steroids and ozone injections. Three male patients who had steroids injection versus two male patients who had ozone injection had a VAS score of 8-9. Six male patients who had steroids injection versus four male patients who had ozone injection had a VAS score of 1-2. Twelve male patients who had steroids injection versus 15 male patients who had ozone injection had a VAS score of 0-1.

Results After Three Months of Steroids Versus Ozone Injection

Figures 3 (A3; B3) show that three male patients who had steroids injection versus two male patients who had ozone injection had a VAS score of 8-9. Nine male patients who had steroids injection versus 11 male patients who had ozone injections had a VAS score of 1-2. Two male patients who had steroids injection versus six male patients who had ozone injections had a VAS score of 0-1. Seven male patients who had steroids injection versus two male patients who had ozone injection had a VAS score of 2-3.

Results After Six Months of Steroids Versus Ozone Injection

Figures 3 (A4; B4) show that three male patients who had steroids injection versus two male patients who had ozone injection had a VAS score of 8-9. Three male patients who had steroids injection versus nine male patients who had ozone injection had a VAS score of 1-2. One male patient who had steroids injection versus four male patients who had ozone injection had a VAS score of 0-1. Fourteen male patients who had steroids injection versus six male patients who had ozone injections had a VAS score of 2-3.

Results Significance for Male Patients for Both Types of Injection After One, Three, and Six Months

We conducted an independent samples t-test (two-tailed, a= 0.05) to assess whether the effect of steroid injection differed significantly from Ozone injection after one month, three months, and six months. The assumptions of normality and equal variances were evaluated with no violations noted. There was no significant difference according to the VAS score scale after one month between the male patients who had steroids injection and the male patients who had ozone injections p= 0.321 > 0.05, and after three months for the same male patients with the type of the same injection with p-value = 0.344 > 0.05. But there is a significant difference in the results between the male patients who had steroids injection and the other males who had ozone injections with p-value = 0.042 < 0.05 after six months of injections (Table 4).

**Table 4:** Variations between ozone and steroids injections in male patients according to VAS score after one, three, and six months of injection N: the total number of male patients equals 21 for each type of injection

	type of injection	N	Mean	Std. Deviation	P-value
VAS score after one month of injection	steroids injection	21	1.57	.746	0.321
	ozone injection	21	1.38	.669	
VAS score after 3 months	steroids injection	21	2.52	.873	0.344
	ozone injection	21	2.00	.894	
VAS score after 6 months	steroids injection	21	2.90	.700	0.042
	ozone injection	21	2.29	.902	

Results according to Mac Nab criteria after one, three, and six months of injection of steroids and ozone in female patients

**Figure 4:**
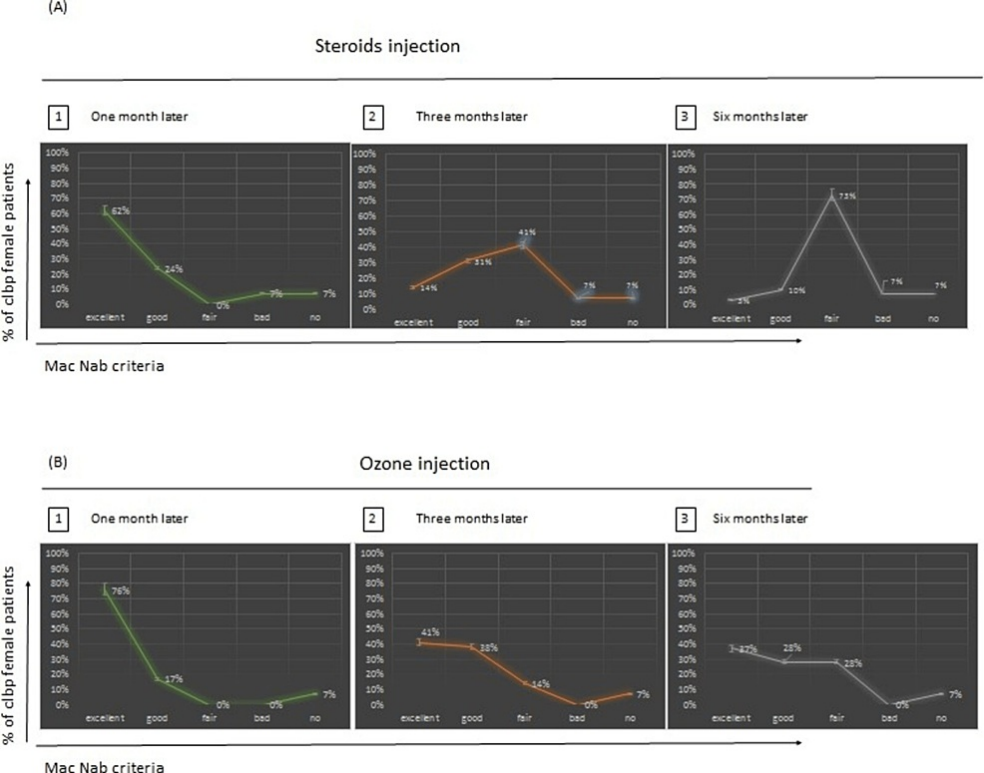
Comparison of steroids and ozone injections after one, three, and six months of treatment according to Mac Nab criteria in female patients. The graph represents the percentage of patients and their response to either type of injection. This response is classified as Excellent, good, fair, bad and no modification, with differences in the results between ozone and steroids injection after one month (p-value: 0.304), three months (p-value: 0.738), and six months (p-value: 0.008).

We evaluated the number of male patients with different Mac Nab criteria after one, three, and six months of injection of steroids and ozone (Figure 4).

Results After One Month of Steroids vs Ozone Injection

Figures 4 (A1; B1) show that 62% of female patients with steroids injection in comparison with 76% of female patients with ozone injections were excellent according to Mac Nab criteria. 24% of female patients with steroids injection in comparison with 17% of female patients with ozone injections were good according to Mac Nab criteria. 7% of female patients with steroids injection in comparison with 0% of female patients with ozone, injections were bad according to Mac Nab criteria. 7% of female patients with steroids injection in comparison with 7% of female patients with ozone injections had no improvement according to Mac Nab criteria.

Results After Three Months of Steroids vs Ozone Injection

Figures 4 (A2; B2) show that 14% of female patients with steroids injection in comparison with 41% of female patients with ozone injections were excellent according to Mac Nab criteria. 31% of female patients with steroids injection in comparison with 38% of female patients with ozone injections were good according to Mac Nab criteria. 41% of female patients with steroid injections in comparison with 14% of female patients with ozone injections were fair according to Mac Nab criteria. 7% of female patients with steroid injections in comparison with 0% of female patients with ozone injections were bad according to Mac Nab criteria. 7% of female patients with steroids injection in comparison with 7% of female patients with ozone injections had no improvement according to Mac Nab criteria.

Results After Six Months of Steroids vs Ozone Injection

Figures 4 (A3; B3) show that 3% of female patients with steroids injection in comparison with 37% of female patients with ozone injections were excellent according to Mac Nab criteria. 10% of female patients with steroids injection in comparison with 28% of female patients with ozone injections were good according to Mac Nab criteria. 73% of female patients with steroid injections in comparison with 28% of female patients with ozone injections were fair according to Mac Nab criteria. 7% of female patients with steroid injections in comparison with 0% of female patients with ozone injections were bad according to Mac Nab criteria. 7% of female patients with steroids injection in comparison with 7% of female patients with ozone injections had no improvement according to Mac Nab criteria.

Differences Between the Results According to Sample T-test in Females After One, Three, and Six Months of Injections Using Mac Nab Criteria

We conducted an independent samples t-test (two-tailed, a= 0.05) to assess whether the effect of steroid injection differed significantly from Ozone injection according to Mac Nab criteria after one month, three months, and six months. The assumptions of normality and equal variances were evaluated with no violations noted. There was no significant difference after one month between the results with female patients who had steroids injection in comparison with the female patients who had Ozone injection with p-value = 0.304 > 0.05, and the same after three months from the injection between Ozone and Steroids between the same female patients with the same type of injections with p-value =0.738 > 0.05, however, there was a significant difference after 6-months of injecting between the results in female patients who had ozone injections and the others females who had steroids injections with p-value =0.008 < 0.05 (Table 5). 

**Table 5:** Variations between ozone and steroids injections in female patients according to Mac Nab criteria after one, three, and six months of injection N= the total number of female patients equals 29 for each type of injection

	type of injection	N*	Mean	Std. Deviation	p-value
Mac Nab criteria after one month	steroids injection	29	1.72	1.222	0.304
	ozone injection	29	1.45	1.055	
Mac Nab criteria after 3 months	steroids injection	29	2.62	1.049	0.738
	ozone injection	29	1.93	1.100	
Mac Nab criteria after 6 months	steroids injection	29	3.03	.778	0.008
	ozone injection	29	2.10	1.145	

Results according to Mac Nab criteria after one, three, and six months of injection of steroids and ozone in male patients

Results After One Month of Steroids vs Ozone Injection

**Figure 5:**
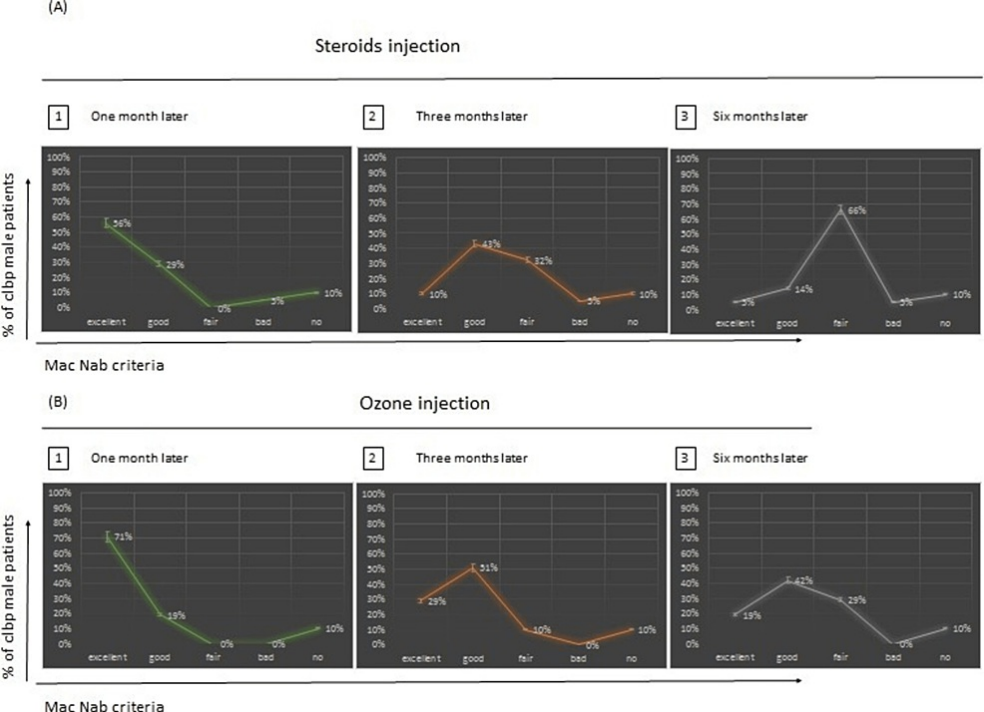
Comparison of steroids and ozone injections after one, three, and six months of treatment according to Mac Nab criteria in male patients. The graph represents the percentage of patients and their response to either type of injection. This response is classified as Excellent, good, fair, bad and no modification, with differences in the results between ozone and steroids injection after one month (p-value: 0.688), three months (p-value: 0.630), and six months (p-value: 0.002).

Figure 5 (A1; B1) shows that 56% of male patients with steroids injection in comparison with 71% of male patients with ozone injections were excellent according to Mac Nab criteria. 29% of male patients with steroids injection in comparison with 19% of male patients with ozone injections were good according to Mac Nab criteria. 5% of male patients with steroids injection in comparison with 0% of male patients with ozone injections were bad according to Mac Nab criteria. 10% of male patients with steroids injection in comparison with 10% of male patients with ozone injections had no improvement according to Mac Nab criteria (Figure 5).

Results After Three Months of Steroids vs Ozone Injection

Figures 5 (A2; B2) show that 10% of male patients with steroids injection in comparison with 29% of male patients with ozone injections were excellent according to Mac Nab criteria. 43% of male patients with steroids injection in comparison with 51% of male patients with ozone injections were good according to Mac Nab criteria. 32% of male patients with steroids injection in comparison with 10% of male patients with ozone injections were fair according to Mac Nab criteria. 5% of male patients with steroids injection in comparison with 0% of male patients with ozone injections were bad according to Mac Nab criteria. 10% of male patients with steroids injection in comparison with 10% of male patients with ozone injections had no improvement according to Mac Nab criteria.

Results After Six Months of Steroids vs Ozone Injection

Figures 5 (A3; B3) show that 5% of male patients with steroids injection in comparison with 19% of male patients with ozone injections were excellent according to Mac Nab criteria. 14% of male patients with steroids injection in comparison with 42% of male patients with ozone injections were good according to Mac Nab criteria. 66% of male patients with steroids injection in comparison with 29% of male patients with ozone injections were fair according to Mac Nab criteria. 5% of male patients with steroids injection in comparison with 0% of male patients with ozone injections were bad according to Mac Nab criteria. 10% of male patients with steroids injection in comparison with 10% of male patients with ozone injections had no improvement according to Mac Nab criteria.

Differences Between the Results According to Sample T-test in Males After One, Three, and Six Months of Injections Using Mac Nab Criteria

We conducted an independent samples t-test (two-tailed, a= 0.05) to assess whether the effect of Steroid injection differed significantly from Ozone injection after one month, three months, and six months. The assumptions of normality and equal variances were evaluated with no violations noted. There was no significant difference according to Mac Nab criteria after one month between the male patients who had steroids injection and the male patients who had ozone injections p= 0.688 >0.05, and after three months for the same male patients with the type of the same injection with p-value= 0.630>0.05. But there is a significant difference in the results among the male patients who had steroids injection and the other males who had ozone injection with p-value=0.002 <0.05 after six months of injection (Table 6).

**Table 6:** Variations between ozone and steroids injections in male patients according to Mac Nab criteria after one, three, and six months of injection N= the total number of female patients equals 21 for each type of injection.

	Type of injection	N*	Mean	Std. Deviation	P-value
Mac Nab criteria after one month	steroids injection	21	1.81	1.289	0.688
	ozone injection	21	1.57	1.207	
Mac Nab criteria after 3 months	steroids injection	21	2.62	1.071	0.630
	ozone injection	21	2.10	1.136	
Mac Nab criteria after 6 months	steroids injection	21	3.00	.894	0.002
	ozone injection	21	2.38	1.117	

Results according to VAS score in relation to radiculopathy for both types of injections

Table 7 shows the number of male patients with L3 radiculopathy with different VAS scores for Ozone and steroids injections before and after one, three, and six months of injection. The total number of males with L3 radiculopathy who had steroid injections is 2. The results before the steroid injection showed that one male patient had a VAS score of (6-7), and another one had a VAS score of (7-8). After one month of steroid injection, two males with L3 radiculopathy had a VAS score of (0-1). After three months of injection, the results were one male patient had a VAS score of (1-2), and the other with a VAS score of (2-3). After six months of steroid injections, the results showed two male patients with VAS score of (2-3). For ozone injection, the results were the same before and after one, three, and six months, with VAS score (8-9) for the only male patient with L3 radiculopathy who had ozone injection (Table 7).

**Table 7:** The number of male patients with L3 radiculopathy with different VAS score for Ozone and steroids before and after one, three, and six months of steroids and ozone injection

	VAS score	Before injection	One month	3 months	6 months	Total number of L3 male patients
Steroids injection	0-1	0	2	0	0	2
	1-2	0	0	1	0	
	2-3	0	0	1	2	
	6-7	1	0	0	0	
	7-8	1	0	0	0	
Ozone injection	8-9	1	1	1	1	1

Table 8 shows the number of female patients with L3 radiculopathy with different VAS score for Ozone and steroids injections, before and after one, three, and six months of injection. The total number of females with L3 who had steroid injections is 6. Before the injection of steroids, there is two female patients who had a VAS score of (6-7), three with a VAS score of (7-8), and only one with a VAS score (of [8-9]). After one month with the same type of injection, the results showed four females with L3 radiculopathy had a VAS score (0-1), one female had a VAS score (1-2) and the other one had a VAS score (8-9). After three months of injection, the results were one female patient had a VAS score of (0-1), four female patients had a VAS score of (2-3), and one female patient had a VAS score of (8-9). After six months of steroid injection, the results showed five female patients with a VAS score of (2-3) and only one female patient had a VAS score of (8-9).

On the other side of the same table, the results showed only one female with L3 radiculopathy had ozone injection, the results were one female with a VAS score (7-8) before injection, become with the same VAS score (0-1) after one, three, and six months of ozone injection (Table 8).

**Table 8:** The number of female patients with L3 radiculopathy with different VAS SCORE for Ozone and steroids before and after one, three, and six months of steroids and ozone injection

	VAS score	Before injection	One month	3 months	6 months	Total number of L3 female patients
Steroids injection	0-1	0	4	1	0	6
	1-2	0	1	0	0	
	2-3	0	0	4	5	
	6-7	2	0	0	0	
	7-8	3	0	0	0	
	8-9	1	1	1	1	
Ozone injection	0-1	0	1	1	1	1
	7-8	1	0	0	0	

Table 9 showed the number of male patients with L4 radiculopathy with different VAS score for Ozone and steroids injections, before and after one, three, and six months of injection. The total number of males with L4 radiculopathy who had steroid injections is 4. The results before the steroid injection showed three male patients had a VAS score (7-8), and only one male patient had a VAS score (8-9). After one month of steroid injection, two males with L4 radiculopathy had a VAS score of (0-1), only one male patient had a VAS score of (1-2), and another one had a VAS score of (8-9). After three months of injection, the results were two male patients had a VAS score (1-2), one male patient had a VAS score (2-3), and only one had a VAS score (8-9). After six months of steroid injections, the results showed one male patient with a VAS score (1-2), two male patients with a VAS score (2-3), and only one male patient with a VAS score (8-9).

The total number of male patients with L4 radiculopathy who had ozone injections is only one patient. The results showed a VAS score of (7-8) before ozone injection, after one, three months, the results one male patient had a VAS score of (1-2), and after six months the results showed one male patient who had VAS score (2-3) (Table 9).

**Table 9:** The number of male patients with L4 radiculopathy with different VAS SCORE for Ozone and steroids before and after one, three, and six months of steroids and ozone injection

	VAS score	Before injection	One month	3 months	6 months	Total number of L4 male patients
Steroids injection	0-1	0	2	0	0	4
	1-2	0	1	2	1	
	2-3	0	0	1	2	
	7-8	3	0	0	0	
	8-9	1	1	1	1	
Ozone injection	0-1	0	0	0	0	1
	1-2	0	1	1	0	
	2-3	0	0	0	1	
	7-8	1	0	0	0	

Table 10 shows the number of female patients with L4 radiculopathy with different VAS score for Ozone and steroids injections, before and after one, three, and six months of injection. The total number of females with L4 who had steroid injections is 6. Before the injection of steroids, there is five female patients who had a VAS score of (7-8), and one female patient with a VAS score of (8-9). After one month with the same type of injection, the results showed four females with L4 radiculopathy had a VAS score of (0-1), and two females had a VAS score of (1-2). After three months of injection, the results were three female patients had a VAS score of (1-2), and three female patients had a VAS score of (2-3). After six months of steroid injections, the results showed only one female patient had a VAS score of (1-2), and five female patients with a VAS score of (2-3) (Table 10).

**Table 10:** The number of female patients with L4 radiculopathy with different VAS score for Ozone and steroids before and after one, three, and six months of steroids and ozone injection

	VAS score	Before injection	One month	3 months	6 months	Total number of L4 female patients
Steroids injection	0-1	0	4	0	0	6
	1-2	0	2	3	1	
	2-3	0	0	3	5	
	7-8	5	0	0	0	
	8-9	1	0	0	0	
Ozone injection	0-1	0	2	1	1	5
	1-2	0	2	1	0	
	2-3	0	0	2	3	
	6-7	2	0	0	0	
	7-8	2	0	0	0	
	8-9	1	1	1	1	

On the other side of the same table, the results showed the results for five females with L4 radiculopathy who had ozone injections. The results were two females with a VAS score (6-7), two female patients with a VAS score (7-8), and only one female patient with a VAS score (8-9) before injection. The results after one month were one female patient with a VAS score (0-1), one female patient with a VAS score (1-2), and 2 female patients with a VAS score (2-3). After three months of ozone injection. Only one female patient had a VAS score of (0-1), another female patient had a VAS score of (1-2), 2 female patients had a VAS score of (2-3), and one female patient had a VAS score of (8-9). After six months the results were one female patient had a VAS score of (0-1), 3 female patients had (2-3), and one female patient had a VAS score of (8-9).

Table 11 shows the number of male patients with L5 radiculopathy with different VAS score for Ozone and steroids injections, before and after one, three, and six months of injection. The total number of males with L5 radiculopathy who had steroid injections is 11. The results before steroid injection showed 4 male patients had a VAS score of (6-7), 4 male patients had (7-8), and 3 male patients had a VAS score of (8-9). After one month of steroid injection, 5 males with L5 radiculopathy had a VAS score of (0-1), 4 male patients had a VAS score of (1-2), and 2 male patients had a VAS score of (8-9). After three months of injection, the results were one male patient had a VAS score of (0-1), two male patients had a VAS score of (1-2), 5 male patients had a VAS score of (1-2), and 2 male patients had a VAS score (8-9). After six months of steroids injection, the results showed one male patient with a VAS score (0-1), 2 male patients with a VAS score (1-2), 6 male patients had (2-3), and 2 male patients with VAS score (8-9). The total number of male patients with L5 radiculopathy who had ozone injections is 6 patients. The results showed 2 male patients with a VAS score of (6-7), and 4 male patients had a VAS score of (7-8) before the ozone injection. After one month the results were 6 male patients had a VAS score of (0-1). After three months the results showed 2 male patients with (0-1), 2 male patients had (1-2), and 2 male patients had a VAS score of (2-3). After six months of ozone injection, the results were one male patient with a VAS score of (0-1), 3 male patients had a VAS score of (1-2), and 2 male patients had a VAS score of (2-3) (Table 11).

**Table 11:** The number of male patients with L5 radiculopathy with different VAS SCORE for Ozone and steroids before and after one, three, and six months of steroids and ozone injection

	VAS score	Before injection	One month	3 months	6 months	Total number of L5 female patients
Steroids injection	0-1	0	5	2	1	11
	1-2	0	4	5	2	
	2-3	0	0	2	6	
	6-7	4	0	0	0	
	7-8	4	0	0	0	
	8-9	3	2	2	2	
Ozone injection	0-1	0	6	2	1	6
	1-2	0	0	2	3	
	2-3	0	0	2	2	
	6-7	2	0	0	0	
	7-8	4	0	0	0	

Table 12 shows the number of female patients with L5 radiculopathy with different VAS score for Ozone and steroid injections, before and after one, three, and six months of injections. The total number of females with L5 who had steroid injections is 5. Before the injections of steroids, there is 2 female patients who had a VAS score of (6-7), 2 female patients of (7-8), and only one female patient with a VAS score of (8-9). After one month with the same type of injections, the results showed 3 females with L5 radiculopathy had a VAS score of (0-1), 1 female had a VAS score of (1-2), and one female patient had a VAS score of (8-9). After three months of injections the results were four female patients had a VAS score of (1-2), 1 female patient had a VAS score of (8-9). After six months of steroid injections, the results showed 2 female patients had a VAS score of (1-2), 2 female patients with a VAS score of (2-3), and only one female patient had a VAS score of (8-9). In the other part of the same table, the results were for 12 females with L5 radiculopathy who had ozone injections. The results were 7 females with A VAS score of (6-7), and 5 female patients with a VAS score of (7-8). The results after one month were 11 female patients with A VAS score of (0-1), and one female patient had a VAS score of (1-2). After three months of ozone injections, 7 female patients had a VAS score of (0-1), and 5 female patients had a VAS score of (1-2). After six months the results were 6 female patients had A VAS score of (0-1), 5 female patients had a VAS score of (1-2), and one female patient had a VAS score of (2-3) (Table 12).

**Table 12:** The number of female patients with L5 radiculopathy with different VAS score for Ozone and steroids before and after one, three, and six months of steroids and ozone injection

	VAS score	Before injection	One month	3 months	6 months	Total number of L5 female patients
Steroids injection	0-1	0	3	0	0	5
	1-2	0	1	4	2	
	2-3	0	0	0	2	
	6-7	2	0	0	0	
	7-8	2	0	0	0	
	8-9	1	1	1	1	
Ozone injection	0-1	0	11	7	6	12
	1-2	0	1	5	5	
	2-3	0	0	0	1	
	6-7	7	0	0	0	
	7-8	5	0	0	0	

Table 13 showed the number of male patients with S1 radiculopathy with different VAS score for Ozone and steroids injections, before and after one, three, and six months of injection. The total number of males with S1 radiculopathy who had steroid injections is 4. The results before the steroid injections showed 1 male patient had a VAS score of (5-6), 2 male patients had a VAS score of (6-7), and 1 male patient had a VAS score of (7-8). After one month of steroid injection, 3 males with S1 radiculopathy had a VAS score of (0-1), only one male patient had a VAS score of (1-2), and 3 male patients had a VAS score of (2-3). After three months of injection, the results were one male patient had a VAS score of (1-2), and 3 male patients had a VAS score of (2-3). After six months of steroid injection, the results showed 4 male patients with a VAS score of (2-3).

The total number of male patients with S1 radiculopathy who had ozone injections is 13 patients. The results showed 2 male patients with a VAS score of (5-6), five male patients had a VAS score of (6-7), five male patients with a VAS score of (7-8), and only one male patient had a VAS score of (8-9) before ozone injection. After one month the results were nine male patients had a VAS score of (0-1), three male patients had a VAS score of (1-2), and one male patient had a VAS score of (8-9). After three months the results showed four male patients with of (0- 1), eight male patients had of (1-2), and only one male patient had a VAS score of (8-9). After six months of ozone injection, the results were three male patients with a VAS score of (0-1), six male patients had a VAS score of (1-2), and three male patients had a VAS score of (2-3), and only one male patient had VAS score of (8- 9) (Table 13).

**Table 13:** The number of male patients with S1 radiculopathy with different VAS score for Ozone and steroids before and after one, three, and six months of steroids and Ozone injection

	VAS score	Before injection	One month	3 months	6 months	Total number of S1 female patients
Steroids injection	0-1	0	3	0	0	4
	1-2	0	1	1	0	
	2-3	0	0	3	4	
	6-7	1	0	0	0	
	7-8	2	0	0	0	
	8-9	1	0	0	0	
Ozone injection	0-1	0	9	4	3	13
	1-2	0	3	8	6	
	2-3	0	0	0	3	
	5-6	2	0	0	0	
	6-7	5	0	0	0	
	7-8	5	0	0	0	
	8-9	1	1	1	1	

Table 14 showed the number of female patients with S1 radiculopathy with different VAS score for Ozone and steroids injections, before and after one, three, and six months of injection. The total number of females with S1 who had steroid injections is 12. Before the injection of steroids, there is nine female patients who had a VAS score of (6-7), one female patient who had (7-8), and 2 female patients with a VAS score of (8-9). After one month with the same type of injection, the results showed seven females with S1 radiculopathy had a VAS score of (0-1), three females had a VAS score of (1-2), and two female patients had a VAS score of (8-9). After three months of injection the results were three female patients had a VAS score of (0-1), two female patients had a VAS score of (1-2), 5 female patients had (2-3), and two female patients had VAS score of (8-9). After six months of steroid injection, the results showed one female patient had a VAS score of (0-1), 9 female patients with a VAS score of (2-3), and 2 female patients had a VAS score of (8-9).

On the other part of the same table, the results were for 11 females with S1 radiculopathy who had ozone injections. The results were 3 females with a VAS score of (5-6), one female patient with a VAS score of (6-7), four female patients had (7-8), and three female patients had a VAS score of (8-9). The results after one month were eight female patients with a VAS score of (0-1), two female patients had a VAS score of (1-2), and only one female patient had a VAS score of (8-9). After three months of ozone injection the results were three female patients had a VAS score of (0-1), five female patients had a VAS score of (1-2), two female patients had (2-3) and only one female patient had a VAS score of (8-9). After six months the results were three female patients had a VAS score of (0-1), three female patients had (1-2), four female patients had a VAS score of (2-3), and only one female patient had a VAS score of (8-9) (Table 14).

**Table 14:** The number of female patients with S1 radiculopathy with different VAS score for Ozone and steroids before and after one, three, and six months of steroids and Ozone injection

	VAS score	Before injection	One month	3 months	6 months	Total number of S1 female patients
Steroids injection	0-1	0	7	3	1	12
	1-2	0	3	2	0	
	2-3	0	0	5	9	
	6-7	9	0	0	0	
	7-8	1	0	0	0	
	8-9	2	2	2	2	
Ozone injection	0-1	0	8	3	3	13
	1-2	0	2	5	3	
	2-3	0	0	2	4	
	5-6	3	0	0	0	
	6-7	1	0	0	0	
	7-8	4	0	0	0	
	8-9	3	1	1	1	

## Discussion

The objective of our study is to assess the benefit of Ozone in comparison with Steroids in CLBP in the short and long term based on Vas score and Mac Nab criteria, both of which are subjective questionnaires. According to our study, 58% of the total number of patients were females and the other 42% were males. This difference between the two genders may be due to females having a lower threshold of perception of pain and differ in reaction to it [32]. In addition, the biological response and physical stress of pregnancy and childbearing, and perimenopausal abdominal weight gain are additional causes for LBP [32].

The objective of our study was to compare the results of injecting either Ozone or steroids as a treatment for CLBP. In the female group, there was no significant difference in the results after one and three months using VAS score (p-values = 0.072 and 0.672, respectively) between steroids and Ozone injections. However, after six months of treatment, there was a significant difference with a p value= 0.001 in females treated with Ozone. Using Mac Nab criteria in the female group, we got the same results as in using the Vas score, i.e., no significant difference was seen after one and three months but with excellent and good results with a significant difference after six months (p-value = 0.008) in those into whom Ozone was injected.

The same results were observed in the male group where no significant difference was seen after one and three months using both Vas score (p values=0.321 and 0.344, respectively) and Mac Nab criteria. After six months of treatment, a significant difference was observed in this group when using the VAS score (p value= 0.042) and excellent and good results with a p value=0.002 using Mac Nab criteria in the Ozone group. This difference in results between the beneficial effect of steroids and the effect of ozone in the short and long term may be due to the anti-inflammatory effect of TFESI that works on the nerve, and hence its function at the disease site [33]. This is an effective tool for the relief of root pain but for a short time [34]. In addition, TFESI is thought to normalize the levels of cytokines and prostaglandins, increase superoxide dismutase levels, minimize reactive oxidant species, and improve local peri-ganglionic circulation with a eutrophic effect on the nerve root [35,36].

The TFEOI works on localized oxygenation and analgesia. Ozone is also important to muscle relaxation and vasodilation as well as reactivation of muscle metabolism, by favoring oxidation of lactate, neutralization of acidosis, enhanced synthesis of adenosine triphosphate, Ca2+ reuptake, and edema reabsorption. Moreover, a further analgesic effect may be derived from the induction of antioxidant enzymes [37]. This may be the cause of the beneficial long-term effect of Ozone rather than steroids that have a different mechanism of action. According to the site of Radiculopathy (rad), the results were different between locations of rad. Those results showed to us the clear benefit of ozone injection in both term short and long term even with different locations of rad. more than steroids which had only benefits in the short term. Plus, as we see the results were more beneficial in the patients who had S1, and L5 radiculopathy than the patients who had L3, and L4 radiculopathy, this may be due to biomechanical reasoning to suspect a difference between spinal levels. When comparing our results with those from different studies, we noticed that we share some similarities.

In a study about the efficacy of TFESI in comparison to epidural Ozone injection, Joel et al. concluded that TFESI is beneficial in the short term and was not as effective in the long term. On the other hand, Ozone was found to be very safe, cheap, and effective as a short-term and long-term solution for patients with back pain along with sciatic nerve pain [38]. M.Gallucci and colleagues performed a study in Italy on 159 patients with lumbar radiculopathy. They divided the patients into two groups. Group A (77 patients) received steroids. Group B (82 patients) received the same dose of steroid with ozone. They found no significant difference between the outcomes of both groups at two weeks and three months (p = 0.72, p = 0.136, respectively). At six months follow-up, the success rate was statistically significant in Group B (74%) compared to Group A (47%) (p < 0.01). They concluded that intraforaminal and intradiscal injections of a steroid plus Ozone is more effective at 6 months than injections of only a steroid in the same sites. They concluded that the Ozone injection has a beneficial effect in the long run [28,36].

Study limitations

Most of the limitations in this study were due to it being a retrospective study. Most of the data were collected from medical files recorded in a subjective manner with many variables missing (past medical history, home medications, smoking, etc.). We did not have the privilege to evaluate real-time imaging examinations. Instead, we examined the previously performed radiological images that were available on the computer system at both sites. We were unable to calculate the incidence of CLBP and lacked follow-up with patients. In addition, the number of new cases was not clear.

Study perspectives

More studies are needed to assess the role of Ozone in treating chronic lower back pain in patients in order to observe if any complication would occur if we increased the number of patients. More retrospective and prospective studies are needed to test whether Ozone injection is better used in the management of chronic lower back pain or if there is another drug that may be of more benefit. They should focus on the causes that drive some Lebanese neurosurgeons to go to surgery quickly when they see a patient with back pain for the first time. Is it their experience? Is it pharmaceutical companies? Is it the government policies? Or it is just related to the patient’s social status? It is important to focus on risk factors of chronic low back pain, and the role of sports and physiotherapy in improving the quality of life, and in decreasing the percentage of people suffering from this medical condition.

## Conclusions

We studied patient profiles of males and females with chronic lower back pain in the Nabatieh area who were treated with either steroids or Ozone injections. According to our study results, the percentage of excellent and good outcomes after one month was approximately the same for both types of injections. Steroids were more beneficial in the short term (1-3 months). Ozone was more beneficial in the long term (six months). Studying patients with different types of radiculopathies, we found that Ozone was better than steroids over both short and long-term durations. These results give us a good plan for the management of increasing morbidity in Lebanese patients. We hope for more studies on the matter to assess the safety of this treatment on a larger scale.

## Figures and Tables

**Figure 6:**
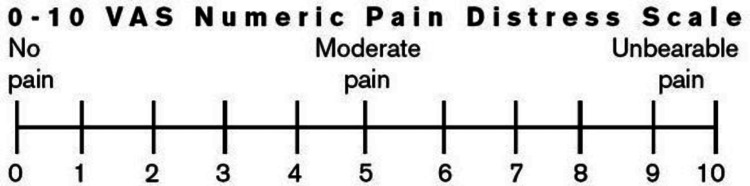
VAS score scale
